# Association between cumulative metabolic score for visceral fat and cardiovascular disease risk in cardiovascular-kidney-metabolic syndrome stage 0–3 patients: a prospective cohort analysis based on CHARLS database

**DOI:** 10.1186/s12872-026-05801-0

**Published:** 2026-03-30

**Authors:** Qiang Chen, Huafeng Fang

**Affiliations:** Mindong Hospital Affiliated to Fujian Medical University, No. 89 Heshan Road, Fu’an City, Ningde, Fujian Province 355000 China

**Keywords:** Cardiovascular-kidney-metabolic syndrome, METS-VF, Cardiovascular disease, Cumulative exposure, Prospective cohort study, CHARLS

## Abstract

**Background and aims:**

The American Heart Association (AHA)'s cardiovascular-kidney-metabolic (CKM) syndrome framework emphasizes prevention during early stages (stages 0-3). The metabolic score for visceral fat (METS-VF) provides accurate cardiometabolic risk assessment, yet studies rely on single-time measurements. We investigated cumulative Metabolic Score for Visceral Fat (cumMETS-VF) exposure and cardiovascular disease (CVD) risk across CKM stages 0-3.

**Methods and results:**

Using the China Health and Retirement Longitudinal Study (CHARLS) 2011–2020, we included 3,341 adults aged ≥45 years with CKM stages 0-3 and no CVD history (mean age 58±9 years, 53.1% female). CumMETS-VF was calculated from 2011-2015, and CVD incidence (coronary heart disease and stroke) was assessed during 2015–2020. Multivariable Cox models, restricted cubic splines (RCS) , and CKM-stratified analyses were performed.

During 60-month follow-up, 634 CVD events (18.9%) occurred. The highest cumMETS-VF quartile showed 42% increased CVD risk versus the lowest (Hazard Ratio(HR) = 1.42, 95% Confidence Interval(CI): 1.11–1.82, P for trend < 0.001). A J-shaped nonlinear association was observed (P-nonlinearity < 0.001). CKM stage significantly modified this association (P for interaction=0.039), with the strongest effect in stage 2 (HR =1.59, 95% CI: 1.16–2.17) but no significance in stages 0-1. Each 1-Standard Deviation(SD) increase in cumMETS-VF conferred 14% higher CVD risk (HR =1.14, 95% CI: 1.06–1.23).

**Conclusion:**

CumMETS-VF exposure showed a J-shaped association with CVD risk in CKM stages 0-3 patients, significantly modified by CKM stage, enabling risk stratification and early intervention.

**Supplementary Information:**

The online version contains supplementary material available at 10.1186/s12872-026-05801-0.

## Introduction

As the global burden of CVD continues to rise, existing prevention and management strategies face significant challenges and require further innovation and refinement [1, 2]. The AHA recently introduced the concept of cardiovascular-kidney-metabolic (CKM) syndrome [3], which integrates metabolic diseases, chronic kidney disease (CKD), and cardiovascular disease into a unified pathophysiological axis, providing a new conceptual foundation for developing an integrated cardiovascular-kidney-metabolic health management approach [4]. Notably, the AHA emphasizes that CVD prevention should focus on the early stages of CKM syndrome (stages 0–3) to enable early identification and proactive intervention in potentially high-risk populations [5].

Obesity, particularly visceral adipose tissue accumulation, is a core driver of cardiometabolic disease [6], and plays a critical role in the development and progression of CKM syndrome [7]. Although numerous large-scale population studies have confirmed a strong association between elevated body mass index (BMI) and clustering of cardiovascular risk factors [8], a substantial proportion of individuals do not fit this pattern [9], This suggests that BMI has limitations in assessing obesity-related cardiovascular risk and fails to accurately reflect visceral fat burden and its metabolic consequences [10, 11].

The metabolic score for visceral fat (METS-VF), a composite indicator integrating the metabolic score for insulin resistance (METS-IR), waist-to-height ratio (WHtR), age, and sex, outperforms traditional metrics in reflecting visceral fat content [12], and is closely associated with CKM syndrome [13], CVD events, and all-cause mortality risk [14].

However, previous studies have predominantly used single-time METS-VF measurements (often baseline values) to assess CKM-related conditions or CVD risk, which inadequately captures the long-term cumulative exposure and dynamic burden of visceral fat metabolism. Epidemiological research has demonstrated that cumulative exposure more accurately reflects the true impact of pathogenic factors on disease occurrence compared to single-time measurements [15–17]. Furthermore, no studies have explored whether the association between cumulative Metabolic Score for Visceral Fat (cumMETS-VF) exposure and CVD risk varies by CKM syndrome stage, which is crucial for implementing stage-specific prevention strategies.

Given these evidence gaps, this study utilized the CHARLS cohort to construct a cumMETS-VF exposure indicator and evaluate its association with CVD risk in populations with CKM stages 0–3, as well as heterogeneity across CKM stages, to provide evidence for early risk stratification.

## Methods

### Study population and design

**Fig. 1:**
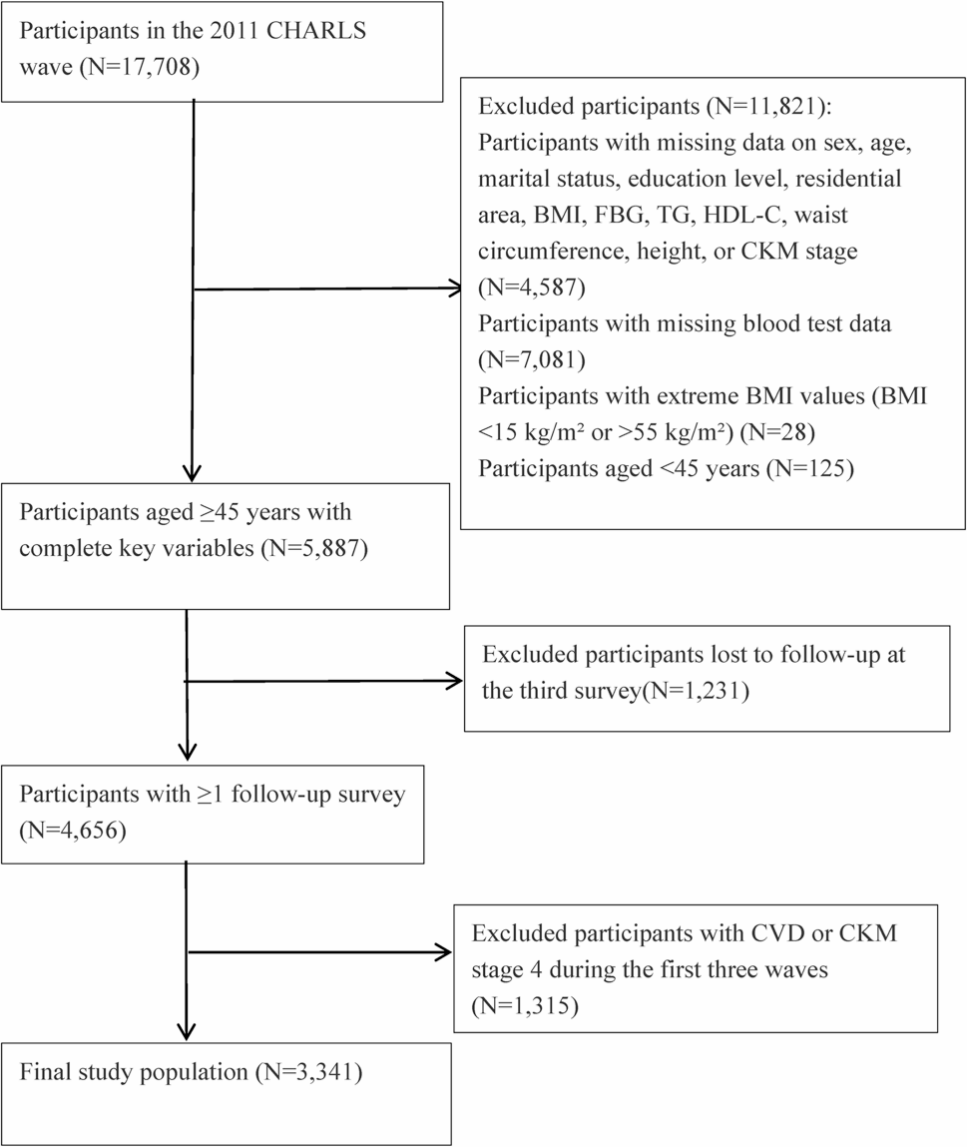
Flowchart of participant selection. Notes:Flow diagram showing the selection of study participants from CHARLS. Of 17,708 baseline participants (2011 wave), 5,887 were included after excluding those aged <45 years (n = 125), with extreme BMI values (n = 28), missing blood test data (n = 7,081), or missing other key variables (n = 4,587). After further excluding participants lost to follow-up (n = 1,231) and those with prevalent CVD or CKM stage 4 (n = 1,315), the final study population comprised 3,341 participants who were stratified by CKM stages 0–3 and followed for CVD incidence through multiple waves

The China Health and Retirement Longitudinal Study (CHARLS) [18] is a nationally representative cohort study conducted by Peking University, targeting community-dwelling residents aged 45 years and older in mainland China. The study employed a multistage stratified probability-proportional-to-size sampling method to recruit participants from urban and rural communities. The baseline survey was launched in 2011, followed by multiple waves of follow-up. Through standardized face-to-face interviews, physical examinations, and fasting blood sample collection [18], information on demographic characteristics, socioeconomic status, health conditions, and lifestyle factors was obtained. This study was a prospective cohort study based on CHARLS, incorporating follow-up data from 2011 to 2020, with a 5-year observation period for CVD. These data are publicly available online at http://charls.pku.edu.cn  [19]. Starting with 17,708 participants from the 2011 baseline survey, the exclusion criteria included: (1) missing data on METS-VF, CKM stage, CVD, or key covariates (complete-case analysis; *n* = 4,587); (2) missing essential blood biochemical indicators (*n* = 7,081); (3) extreme BMI values (< 15 or > 55 kg/m²) (*n* = 28); (4) age < 45 years (*n* = 125). Among the remaining 5,887 participants, those lost to follow-up at the third wave (*n* = 1,231) and those who reported cardiovascular disease or were in CKM stage 4 between baseline and the third wave (*n* = 1,315) were further excluded. Ultimately, 3,341 participants in CKM stages 0–3 were included in the analysis (Fig. 1). To assess potential selection bias introduced by exclusions at baseline, we compared baseline characteristics between participants included in the final analysis and those excluded at baseline, who were primarily excluded due to missing data on key variables (Table S1).

This study was conducted in accordance with the ethical principles outlined in the Declaration of Helsinki and was approved by the Biomedical Ethics Review Committee of Peking University (IRB00001052-11015). Written informed consent was obtained from all participants.

### Exposure

The exposure variable in this study was METS-VF, calculated using the following formula [12]:

$$\begin{aligned} \mathrm{METS}-\mathrm{VF}=&\:4.466+0.011\left[\left(\mathrm{In}\left(\mathrm{METS}-\mathrm{IR}\right)\right)3\right]\\&+3.239\left[\left(\mathrm{In}\;\left(\mathrm{WHtR}\right)\right)3\right]+0.319\;\left(\mathrm{sex}\right)\\&+0.594\left(\mathrm{In}\left(\mathrm{age}\right)\right) \end{aligned}$$

METS-IR was calculated as [20]:

$$\mathrm{METS}-\mathrm{IR}=\mathrm{In}\left(\frac{(2\ast\mathrm{fasting}\;\mathrm{glucose}+\mathrm{fasting}\;\mathrm{triglycerides})\times\mathrm{BMI}}{\mathrm{high}-\mathrm{density}\;\mathrm{lipoprotein}\;\mathrm{cholesterol}}\right)$$

WHtR was calculated as the waist circumference-to-height ratio [21].

CumMETS-VF was defined as the average METS-VF value between each pair of consecutive surveys multiplied by the time interval between these two consecutive surveys, calculated using the following formula [22, 23]:

$$\begin{aligned} \mathrm{CumMETS}-\mathrm{VF}=& \left(2012\;\mathrm{METS}-\mathrm{VF}+2015\mathrm{METS}-\mathrm{VF}\right)\\&/2\times\mathrm{follow}-\mathrm{up}\;\mathrm{years}\left(2015-2012\right) \end{aligned}$$

where 2012 METS-VF and 2015 METS-VF represent the METS-VF values measured in 2012 and 2015, respectively, and (2015–2012) represents the follow-up interval in years.

### Outcome

The primary outcome of this study was the first occurrence of CVD during the follow-up period, including incident coronary heart disease (myocardial infarction, angina, or other self-reported physician-diagnosed coronary heart disease) and stroke. Participants were followed up for 5 years from baseline, and outcome ascertainment was primarily based on standardized questions in the follow-up questionnaire: “Have you ever been told by a doctor that you have had a myocardial infarction/angina/coronary heart disease/stroke?” [24].

### Definition of CKM syndrome stages 0–3

According to the AHA CKM syndrome staging criteria [3], participants were classified into stages 0–3: Stage 0, no CKM risk factors; Stage 1, excess or dysfunctional adiposity; Stage 2, metabolic risk factors and/or CKD; Stage 3, subclinical CVD in CKM. The operational definition of subclinical cardiovascular disease was established based on risk-equivalent criteria, including a predicted 10-year cardiovascular disease risk ≥ 20% using the PREVENT Eqs. [5, 25], or very high-risk CKD stage as defined by KDIGO criteria [26]. Detailed staging criteria, metabolic syndrome definitions, and risk prediction equations are provided in Supplementary Tables S2, S3, and S4.

The estimated glomerular filtration rate (eGFR) was calculated using the Chronic Kidney Disease Epidemiology Collaboration (CKD-EPI) Eq. [27]. The formula is as follows:

$$\mathrm{eGFR}=141\times\mathrm{min}\left(\frac{\mathrm{Scr}}{\kappa,1}\right)^{\alpha}\times\mathrm{max}\left(\frac{\mathrm{Scr}}{\kappa,1}\right)^{-1.209}\times0.993^{\mathrm{age}}\times\mathrm{sex}\;\mathrm{factor}$$

Where Scr is serum creatinine (mg/dL); κ and α are sex-specific parameters, with values of 0.7 and − 0.329 for females, and 0.9 and − 0.411 for males, respectively; the sex coefficient is 1.018 for females and 1.0 for males. Since the study population consisted entirely of Chinese individuals (non-Black race), the race correction factor of 1.159 was not applied. The unit for eGFR is mL/min/1.73 m².

### Data collection

The variables included in this study were:

1)Baseline demographic characteristics, including sex, age, marital status, educational level, and residential area; 2) Lifestyle-related variables, including smoking status and alcohol consumption habits; 3) Physical examination parameters, including height, waist circumference, WHtR, BMI, Systolic Blood Pressure(SBP), and Diastolic Blood Pressure(DBP); 4) Clinical history and treatment status, including prevalence and medication use for dyslipidemia, hypertension, diabetes, kidney disease, and liver disease; 5) Laboratory covariates, including high-sensitivity C-reactive protein (Hs-CRP), HbA1C, Glucose(GLU), Low-Density Lipoprotein Cholesterol(LDL-C), High-Density Lipoprotein Cholesterol(HDL-C), Total Cholesterol(TC), Triglyceride(TG), Scr, Blood Urea Nitrogen(BUN), Uric Acid(UA), and GFR.

Participants were classified into the hypertension group if they met any of the following criteria: history of hypertension diagnosis or treatment, or SBP ≥ 130 mmHg and/or DBP ≥ 80 mmHg at enrollment. Diabetes was defined as self-reported diabetes history or HbA1C ≥ 6.5% at enrollment. Dyslipidemia was defined as previous confirmed diagnosis, or lipid levels meeting any of the following: TG ≥ 2.3 mmol/L, TC ≥ 6.2 mmol/L, and/or LDL-C ≥ 4.1 mmol/L.

### Statistical analysis

We assessed the normality of all continuous variables. Normally distributed data were presented as mean ± standard deviation (SD), while skewed data were expressed as median Interquartile Range (IQR). Categorical variables were presented as frequencies and percentages. Baseline characteristics were grouped according to cumMETS-VF quartiles (Q1-Q4). Between-group comparisons for categorical variables were performed using Pearson’s chi-square test (with simulated P-values), while normally distributed continuous variables were compared using one-way analysis of variance (ANOVA), and non-normally distributed variables were analyzed using the Kruskal-Wallis rank-sum test.

Cox proportional hazards regression models were used to examine the association between cumMETS-VF exposure (quartiles) and incident CVD risk. Three progressively adjusted models were fitted: Model 1 adjusted for age and sex; Model 2 additionally adjusted for marital status, education, residential area, smoking, drinking, systolic blood pressure, diastolic blood pressure, fasting blood glucose, and HbA1c; Model 3 further adjusted for total cholesterol, HDL-C, eGFR, history of dyslipidemia, hypertension and diabetes, and medications for dyslipidemia, hypertension and diabetes. These additional adjustments were included to provide a more conservative estimate of the association. Although some of these variables may lie on the potential causal pathway between visceral fat and CVD, their inclusion allows us to assess whether the observed association is independent of these clinically relevant conditions. Hazard ratio (HRs) with 95% confidence intervals (CIs) were reported, and a test for trend across quartiles was performed. The proportional hazards assumption was assessed using Schoenfeld residuals. Multicollinearity was evaluated using variance inflation factors (VIFs). CumMETS-VF was categorized into quartiles (Q1-Q4), with Q1 as the reference, and HR and 95% CI were calculated for each group, with linear trend tests performed (P for trend). CumMETS-VF was standardized and treated as a continuous variable to calculate HR per 1-SD increase. Restricted cubic spline (RCS) analysis was used to evaluate the dose-response relationship between cumMETS-VF and CVD risk, with 4 knots (5th, 35th, 65th, 95th percentiles) set for the overall population analysis and 3 knots (10th, 50th, 90th percentiles) for CKM stage-stratified analysis, using the median cumMETS-VF as the reference (HR = 1.0). The overall association (P for overall) and nonlinearity (P for nonlinearity) were evaluated by likelihood ratio tests. Kaplan-Meier survival curves were plotted, and log-rank tests were used to compare between-group differences.

To assess the robustness of the results, we conducted subgroup analyses and sensitivity analyses. Subgroup analyses were performed by age (< 60 vs. ≥60 years), sex, smoking status, alcohol consumption status, baseline disease status (hyperlipidemia, hypertension, diabetes), and CKM stage (stages 0–3), with effect modification evaluated by adding interaction terms in Cox models (P for interaction). RCS analyses were conducted separately in the overall population and within each CKM stage population. Sensitivity analyses excluded participants with baseline cancer history or those using diabetes medications/insulin, and repeated the main analyses.

All analyses were performed using R software (version 4.4.1, https://www.R-project.org). All hypothesis tests were two-sided, and *P* < 0.05 was considered statistically significant.

## Results

### Baseline characteristics of study participants

This study included 3,341 participants with CKM syndrome stages 0–3 (53.1% female, mean age 58 ± 9 years). During a mean follow-up of 60 months, 634 incident CVD events were recorded. According to cumMETS-VF quartiles (Table 1), Q4 (high exposure) participants were older, had a higher proportion of urban residents, and lower proportions of smoking and alcohol consumption. Compared with Q1, Q4 demonstrated higher obesity indices (BMI 27.0 ± 3.2 vs. 20.5 ± 3.0 kg/m²; WHtR 0.61 ± 0.05 vs. 0.46 ± 0.08), inflammatory levels (Hs-CRP 1.40 vs. 0.76 mg/L), dyslipidemia, GLU metabolism disorders, and reduced renal function. The prevalence of dyslipidemia, hypertension, and diabetes in Q4 was significantly higher than in Q1 (15%, 38%, 10% vs. 4.2%, 11%, 3.1%, respectively), with correspondingly increased medication use. CKM stage increased progressively with cumulative exposure (P for trend < 0.001). All P-values were < 0.05. No evidence of multicollinearity was observed among the covariates (Table S5). Although some covariates exhibited potential time-dependent effects, Schoenfeld residual analyses indicated that the proportional hazards assumption was not violated at the global level (Table S6).

**Table 1:** Baseline Characteristics of Participants With CKM Syndrome Stages 0–3: Stratified by Quartiles of cumMETS-VF Exposure

Characteristic	Overall *N* = 3,341	Q1 *N* = 837	Q2 *N* = 844	Q3 *N* = 831	Q4 *N* = 829	*p*-value
Male, *n* (%)	1,566 (47)	443 (53)	369 (44)	334 (40)	420 (51)	< 0.001
Age(years)	58 ± 9	57 ± 9	57 ± 8	58 ± 9	60 ± 9	< 0.001
Married, *n* (%)	2,904 (87)	730 (87)	742 (88)	711 (86)	721 (87)	0.5
Educational attainment, *n* (%)						0.4
Elementary school or below	2,354 (70)	592 (71)	584 (69)	596 (72)	582 (70)	
Middle school	910 (27)	231 (28)	238 (28)	220 (26)	221 (27)	
College or above	77 (2.3)	14 (1.7)	22 (2.6)	15 (1.8)	26 (3.1)	
Urban residence, *n* (%)	448 (13)	81 (9.7)	103 (12)	110 (13)	154 (19)	< 0.001
BMI(kg/m²)	23.4 ± 3.6	20.5 ± 3.0	22.1 ± 2.0	24.2 ± 2.5	27.0 ± 3.2	< 0.001
WHtR(cm)	0.53 ± 0.08	0.46 ± 0.08	0.51 ± 0.03	0.56 ± 0.04	0.61 ± 0.05	< 0.001
smoking status, *n* (%)						< 0.001
Current Smoker	1,031 (31)	332 (40)	257 (30)	215 (26)	227 (27)	
Never Smoked	2,064 (62)	455 (54)	541 (64)	561 (68)	507 (61)	
Former Smoker	246 (7.4)	50 (6.0)	46 (5.5)	55 (6.6)	95 (11)	
drinking status, *n* (%)						0.002
Current Drinker	1,088 (33)	291 (35)	286 (34)	238 (29)	273 (33)	
Never Drinker	2,004 (60)	483 (58)	513 (61)	534 (64)	474 (57)	
Former Drinker	249 (7.5)	63 (7.5)	45 (5.3)	59 (7.1)	82 (9.9)	
Dyslipidaemia, *n* (%)	272 (8.1)	35(4.2)	50 (5.9)	66 (7.9)	121 (15)	< 0.001
Hypertension, *n* (%)	714 (21)	90 (11)	129 (15)	178 (21)	317 (38)	< 0.001
Diabetes, *n* (%)	186 (5.6)	26 (3.1)	32 (3.8)	43 (5.2)	85 (10)	< 0.001
Diabetes medication use, *n* (%)	113 (3.4)	18 (2.2)	20 (2.4)	27 (3.2)	48 (5.8)	< 0.001
Diabetes insulin use, *n* (%)	16 (0.5)	3 (0.4)	8 (0.9)	3 (0.4)	2 (0.2)	0.2
Kidney disease, *n* (%)	138 (4.1)	39 (4.7)	32 (3.8)	35 (4.2)	32 (3.9)	0.8
Liver disease, *n* (%)	91 (2.7)	29 (3.5)	22 (2.6)	21 (2.5)	19 (2.3)	0.5
Lipid-lowering medication use, *n* (%)	133 (4.0)	14 (1.7)	19 (2.3)	33 (4.0)	67 (8.1)	< 0.001
Antihypertensive medication use, *n* (%)	488 (15)	61 (7.3)	70 (8.3)	119 (14)	238 (29)	< 0.001
Hs-CRP (mg/L)	0.98 (0.54–1.98)	0.76 (0.44–1.58)	0.77 (0.47–1.56)	1.03 (0.59–2.10)	1.40 (0.76–2.59)	< 0.001
LDL­C(mg/dL)	117 ± 35	113 ± 32	115 ± 34	121 ± 36	120 ± 37	< 0.001
HDL­C(mg/dL)	52 ± 15	58 ± 16	54 ± 15	49 ± 14	45 ± 12	< 0.001
TC(mg/dL)	194 ± 38	190 ± 37	191 ± 38	198 ± 38	199 ± 38	< 0.001
TG(mg/dL)	127 ± 93	97 ± 58	110 ± 73	136 ± 94	166 ± 121	< 0.001
GLU (mg/dL)	109 ± 33	105 ± 31	105 ± 27	110 ± 34	117 ± 39	< 0.001
HbA1c, %	5.28 ± 0.83	5.17 ± 0.70	5.20 ± 0.77	5.28 ± 0.87	5.47 ± 0.92	< 0.001
Scr (mg/dL)	0.77 ± 0.18	0.76 ± 0.17	0.75 ± 0.17	0.76 ± 0.19	0.79 ± 0.19	< 0.001
BUN (mg/dL)	15.6 ± 4.3	16.1 ± 4.6	15.5 ± 4.3	15.3 ± 4.3	15.7 ± 4.2	0.013
UA (mg/dL)	4.34 ± 1.19	4.18 ± 1.12	4.15 ± 1.10	4.32 ± 1.17	4.72 ± 1.29	< 0.001
GFR (mL/min/1.73 m²)	94 ± 14	95 ± 13	96 ± 13	93 ± 13	91 ± 14	< 0.001
CKM						< 0.001
0	360 (11)	233 (28)	106 (13)	17 (2.0)	4 (0.5)	
1	843 (25)	213 (25)	251 (30)	246 (30)	133 (16)	
2	1,789 (54)	345 (41)	457 (54)	487 (59)	500 (60)	
3	349 (10)	46 (5.5)	30 (3.6)	81 (9.7)	192 (23)	

### Association between CumMETS-VF exposure and CVD in patients with CKM Syndrome stages 0–3

**Fig. 2:**
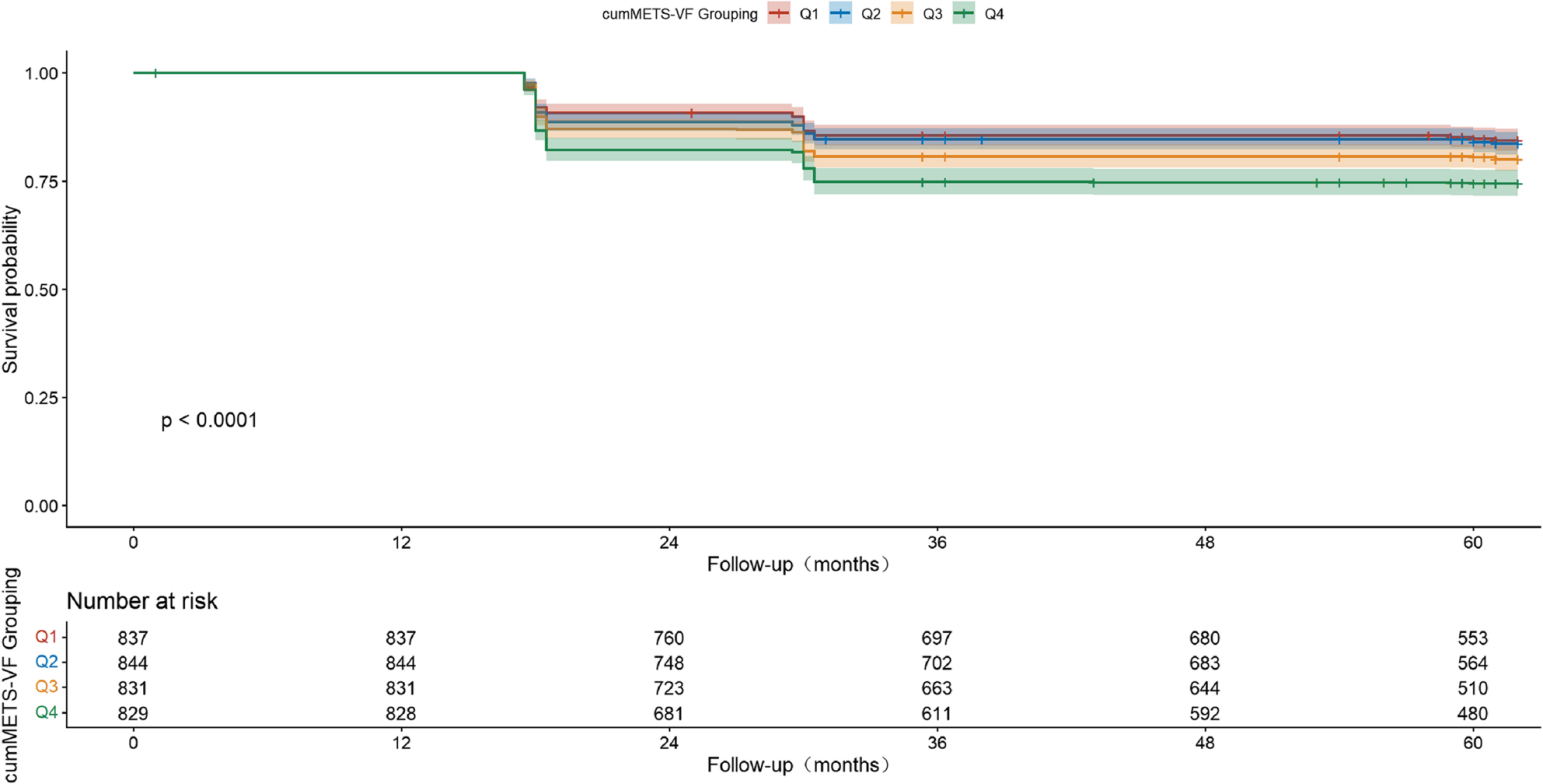
Kaplan-Meier Survival by CumMETS-VF Quartiles in CKM Stages 0–3. Notes:Kaplan-Meier curves showing cumulative incidence of cardiovascular disease (CVD) across quartiles of cumMETS-VF during follow-up period. Q1 represents the lowest quartile and Q4 the highest quartile of cumMETS-VF. The number at risk at each time point is shown below the x-axis. Log-rank test: *P *< 0.0001

Kaplan-Meier survival curves showed that CVD-free survival rates exhibited a gradient decline with increasing cumMETS-VF quartiles (Q1 to Q4) (Log-rank *P* < 0.0001; Fig. 2). At the end of follow-up, cumulative survival rates for Q1-Q4 groups were 84.8%, 84.1%, 80.5%, and 74.6%, respectively.

Cox regression analysis results are presented in Table 2. In the fully adjusted model (Model 3), participants in the highest cumulative METS-VF quartile (Q4) had a significantly higher risk of incident CVD than those in Q1 (HR = 1.33, 95% CI: 1.04–1.72). No significant associations were observed for Q2 (HR = 1.00, 95% CI: 0.79–1.28) or Q3 (HR = 1.12, 95% CI: 0.88–1.43). A significant dose–response pattern was supported by the trend test (P for trend < 0.001). The Q4 association remained directionally consistent after adjustment, with attenuation from Model 1 (HR = 1.71) to Model 2 (HR = 1.53) and Model 3 (HR = 1.33).

**Table 2:** Association of Cumulative Exposure to METS-VF with the Risk of CVD in a CKM Syndrome Stage 0–3 Population

		**HR (95% CI)**		
Quartile	Cases/Total	Model 1	Model 2	Model 3
Q1 (Ref)	127 / 837	Reference	Reference	Reference
Q2	134 / 844	1.03(0.81,1.32)	1.01(0.79,1.29)	1.00(0.79, 1.28)
Q3	162 / 831	1.26(1.00, 1.59)	1.19(0.94, 1.51)	1.12(0.88, 1.43)
Q4	211 / 829	1.71(1.37, 2.14)	1.53(1.21, 1.94)	1.33(1.04, 1.72)
P for trend		<0.001	<0.001	<0.001
Events/*N*	634/3341			

Model 1: Adjusted for age and sex

Model 2: Model 1 + marital status, education, residential area, smoking, drinking, systolic blood pressure, diastolic blood pressure, fasting blood glucose, and HbA1c

Model 3: Model 2 + total cholesterol, HDL-C, eGFR, history of dyslipidemia, hypertension, diabetes, and medications for dyslipidemia, hypertension, and diabetes

Events/*N*: Number of CVD events / Total number of participants

**Fig. 3:**
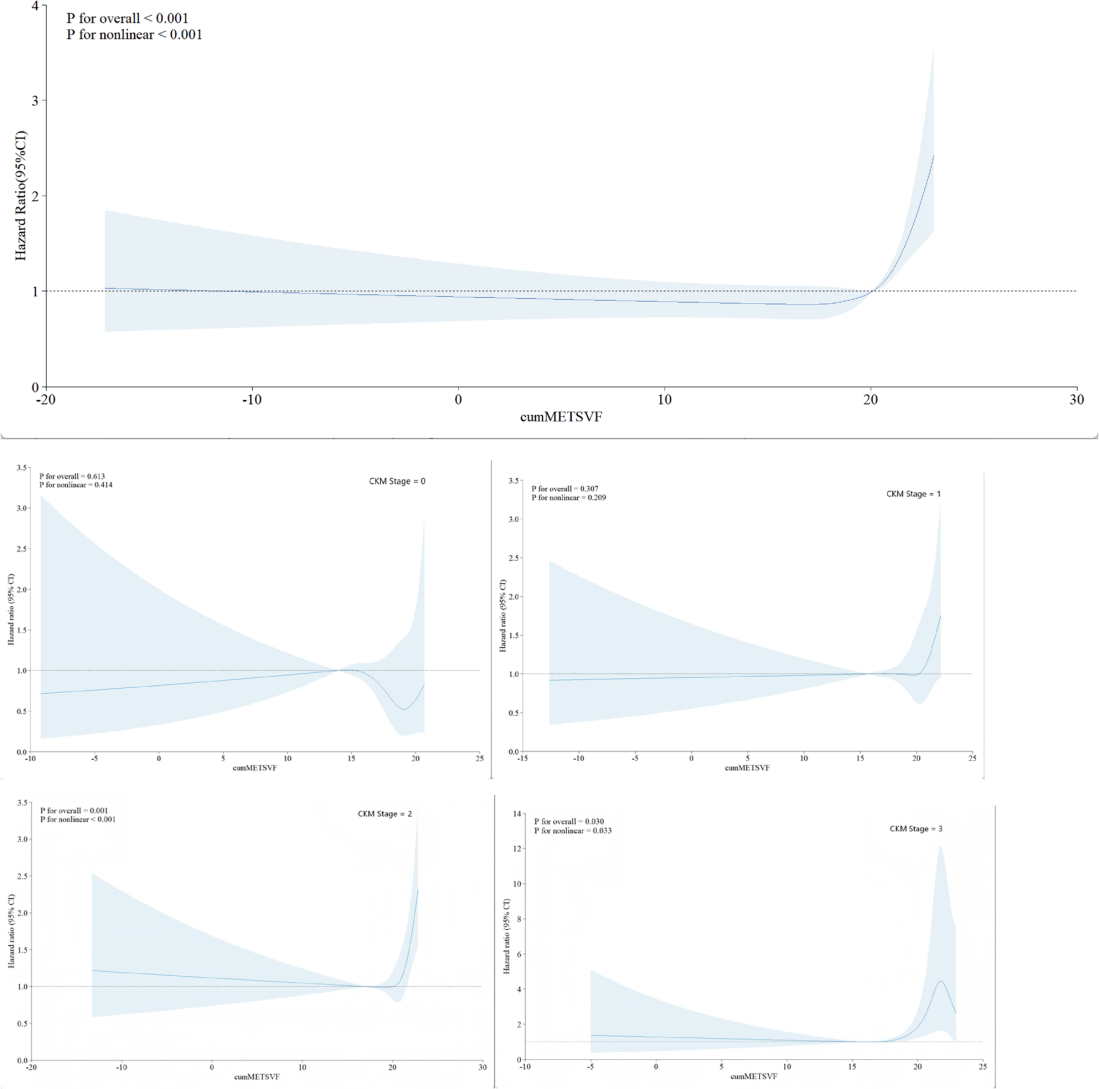
Dose-Response of CumMETS-VF and CVD Risk by CKM Stage. Note: Restricted cubic spline curves with 4 knots (overall) or 3 knots (stage-specific). Reference: HR = 1.0 at median cumMETS-VF. Solid lines: HRs; shaded areas: 95% CIs. P values indicate overall association and non-linearity. Significant associations observed in Stages 2, 3, and overall (P < 0.05). Y-axis for Stage 3 (0–14) differs from other panels due to elevated HRs

Furthermore, restricted cubic spline analysis revealed a significant J-shaped nonlinear association between cumMETS-VF exposure and CVD risk (Fig. 3, P for overall < 0.001; P for nonlinearity < 0.001), with risk beginning to rise beyond approximately 20. Stage-stratified analysis (3 knots at 10th, 50th, 90th percentiles) demonstrated significant heterogeneity in dose-response patterns across different CKM stages: Stages 0 and 1 showed non-significant overall associations (P for overall = 0.613 and 0.307, respectively), with relatively flat curves; Stage 2 displayed a significant nonlinear association (P for overall = 0.001, P for nonlinearity < 0.001), with risk beginning to rise when cumMETS-VF exceeded approximately 20; Stage 3 showed a significant association (P for overall = 0.030, P for nonlinearity = 0.033), with HR declining at moderate exposure levels (cumMETS-VF approximately 10–20) and exhibiting dramatic fluctuations in the high exposure zone (cumMETS-VF > 20), with markedly widened CIs.

### Subgroup analysis

The association between cumMETS-VF exposure and CVD risk remained consistent across most subgroups (Table 3). In the overall population, each 1-SD increase in cumMETS-VF was associated with a 14% increased CVD risk (HR = 1.14, 95% CI: 1.06–1.23, *P* = 0.001). Stratified analyses showed that age, sex, smoking, alcohol consumption, dyslipidemia, and hypertension did not significantly modify the association between cumMETS-VF and CVD risk (all P for interaction > 0.05). The association was more pronounced among non-diabetic individuals (HR = 1.15, 95% CI: 1.07–1.25, *P* < 0.001), although diabetes status did not produce significant interaction (*P* = 0.192). Stratified analysis by CKM stage revealed significant heterogeneity (P for interaction = 0.039), with the association being most prominent in CKM stage 3 (HR = 1.59, 95% CI: 1.16–2.17, *P* = 0.004), while the associations were non-significant in CKM stage 0 (HR = 1.09, 95% CI: 0.61–1.96, *P* = 0.774), CKM stage 1 (HR = 1.11, 95% CI: 0.95–1.31, *P* = 0.195), and CKM stage 2 (HR = 1.08, 95% CI: 0.98–1.19, *P* = 0.126).

**Table 3:** Subgroup Analyses of the Association Between Cumulative Exposure to METS-VF and the Risk of CVD in a CKM Syndrome Stage 0–3 Population

Variable		Count	Percent(%)	HR (95% CI)	*P* value	*P* for interaction
Overall		3341	100	1.14 (1.06–1.23)	0.001	-
Age	< 60	1975	59.1	1.14 (1.03–1.27)	0.012	0.551
	≥ 60	1366	40.9	1.13 (1.02–1.25)	0.025	-
Gender	Females	1775	53.1	1.12 (1.01–1.24)	0.026	0.643
	Males	1566	46.9	1.15 (1.03–1.29)	0.011	-
Smoking	Current Smoker	1031	30.9	1.11 (0.97–1.27)	0.144	0.919
	Former Smoker	246	7.4	1.20 (0.91–1.57)	0.193	-
	Never Smoked	2064	61.8	1.14 (1.04–1.26)	0.005	-
Drinking	Current Drinker	1088	32.6	1.17 (1.02–1.34)	0.024	0.762
	Former Drinker	249	7.5	1.09 (0.86–1.37)	0.492	–
	Never Drinker	2004	60.0	1.14 (1.03–1.25)	0.009	-
Dyslipidemia	No	3069	91.9	1.14 (1.05–1.24)	0.001	0.886
	Yes	272	8.1	1.11 (0.90–1.37)	0.333	-
Hypertension	No	2627	78.6	1.11 (1.02–1.21)	0.014	0.519
	Yes	714	21.4	1.21 (1.04–1.40)	0.014	-
Diabetes	No	3155	94.4	1.15 (1.07–1.25)	< 0.001	0.192
	Yes	186	5.6	1.00 (0.76–1.33)	0.976	-
CKM	0	360	10.8	1.09 (0.61–1.96)	0.774	0.039
	1	843	25.2	1.11 (0.95–1.31)	0.195	-
	2	1789	53.5	1.08 (0.98–1.19)	0.126	-
	3	349	10.4	1.59 (1.16–2.17)	0.004	-

### Sensitivity analysis

In sensitivity analyses, we excluded participants with a baseline history of cancer (Figure S1; Figure S2; Table S7; Table S8) or those using diabetes medications/insulin (Figure S3; Figure S4; Table S9; Table S10), and the association between cumMETS-VF and CVD risk remained robust and consistent. After excluding cancer cases (*n* = 3,283, 625 CVD events), Q4 still showed significantly elevated CVD risk (HR = 1.42, 95% CI: 1.11–1.82, P for trend < 0.001). The association in CKM stage 3 was further strengthened (HR = 1.61, 95% CI: 1.18–2.20, *P* = 0.003), with more pronounced interaction (P for interaction = 0.026). After excluding diabetes medication users (*n* = 3,038, 544 CVD events), main results remained consistent (Q4: HR = 1.42, 95% CI: 1.11–1.82), but the CKM stage interaction was attenuated (P for interaction = 0.14).

## Discussion

In this prospective cohort of 3,341 Chinese middle-aged and older adults with CKM stages 0–3 (median follow-up, 60 months), higher cumMETS-VF was associated with an increased 5-year risk of incident CVD [28]. This association was consistent across Kaplan–Meier, fully adjusted Cox, and restricted cubic spline analyses, which showed a J-shaped dose–response pattern with higher risk at elevated exposure levels. Stratified analyses indicated that the association was mainly present in CKM stage 3, but not statistically significant in stages 0–1. Notably, some covariates in Model 3 may be mediators, suggesting the fully adjusted estimates are conservative.

A possible explanation for the J-shaped relationship is multifactorial: preserved metabolic homeostasis at low exposure, and inflammation, insulin resistance, and neurohormonal activation at high exposure, while the intermediate plateau may reflect survivor bias [29, 30]. METS-VF integrates METS-IR, WHtR, age, and sex, providing a more accurate assessment of visceral fat burden than BMI [12, 31, 32]. This study is the first to evaluate cumMETS-VF exposure on CVD risk across CKM stages 0–3, revealing that the cumulative effect intensifies with advancing stage and peaks at stage 3. Mechanistically, visceral adiposity may increase CVD risk through chronic inflammation promoting atherosclerosis (hs-CRP: 1.40 vs. 0.76 mg/L, *P* < 0.001) [33, 34], activation of the Renin-Angiotensin-Aldosterone System (RAAS) leading to insulin resistance and endothelial dysfunction [33–35], and cumulative dose-dependent vascular damage [16, 36]. The stronger association in CKM stage 3 supports a potential “second-hit” effect, whereby excess visceral fat burden exacerbates subclinical cardiovascular dysfunction. Notably, although CKM stage modified the association between cumMETS-VF and CVD risk, this interaction disappeared after excluding participants on glucose-lowering medications, suggesting a potential role of diabetes and its treatment. This finding highlights the complex interplay among CKM stage, glycemic status, and visceral fat, which warrants further investigation.

Compared with previous studies, this study demonstrates significant innovation. First, while previous studies primarily used single-measurement METS-VF to assess CVD risk [14, 37, 38], this study is the first to construct a cumMETS-VF indicator in CKM stages 0–3 populations, capturing long-term metabolic burden through multiple time-point measurements, which more comprehensively reflects the true impact of pathogenic factors compared to single measurements [16]. Subgroup analyses showed broadly consistent associations across demographic and clinical strata; however, effect heterogeneity emerged across CKM stages, with a stronger association in advanced stages, particularly stage 3. This pattern is consistent with the AHA’s proposed strategy of prioritizing upstream interventions in earlier CKM stages [3] and supports a stage-stratified, individualized prevention approach. Restricted cubic spline analysis indicated a nonlinear dose–response relationship, with risk increasing notably beyond cumMETS-VF ≈ 20. Sensitivity analyses excluding participants with baseline cancer and those using glucose-lowering medications or insulin yielded materially similar results, supporting the robustness of our findings. Based on the above evidence, this study provides the following clinical implications for precision management of CKM syndrome: cumMETS-VF can serve as a dynamic monitoring indicator for cardiovascular risk; the risk threshold identified in this study (cumMETS-VF ≈ 20) can be used for stratified management, with lifestyle interventions as the primary approach when cumMETS-VF < 20, while those with cumulative exposure ≥ 20 require intensified pharmacological treatment and close monitoring; CKM stage 3 patients are more sensitive to cumulative metabolic exposure and should be prioritized as high-risk populations for intervention, whereas CKM stages 0–2 populations should focus on early monitoring of cumMETS-VF with preventive management initiated before reaching the risk threshold.

This study has several limitations. First, CVD outcomes were based on self-reported physician diagnoses in the CHARLS database, which may be subject to recall and misclassification bias. Second, METS-VF was calculated from routine physical examinations and laboratory measures rather than direct assessment of visceral fat. Third, residual confounding (such as genetic and dietary factors) may still exist. In addition, substantial baseline exclusions due to missing data may have introduced selection bias, although this was evaluated using baseline comparisons. Fourth, the study population was limited to Chinese individuals aged ≥ 45 years, and the generalizability of the findings requires further validation. Fifth, in the high exposure range (cumMETS-VF > 20), CIs for CKM stages 0 and 3 were wide, and the relatively small sample size in CKM stage 3 may have affected estimate stability; therefore, these results should be interpreted with caution. Sixth, CKM stage 3 was defined using PREVENT-predicted 10-year CVD risk ≥ 20%, a proxy for subclinical disease that may introduce misclassification.

Future research should (1) delineate mechanistic pathways linking visceral adiposity to cardiovascular risk across CKM stages (2), validate the predictive performance of cumMETS-VF in non-Asian and younger populations, and (3) determine whether interventions that reduce visceral adiposity and cumulative exposure translate into lower subsequent CVD risk.

## Conclusions

This prospective cohort study found that cumMETS-VF exposure demonstrates a J-shaped nonlinear association with CVD risk in patients with CKM syndrome stages 0–3, and this association is significantly modified by CKM stage. CumMETS-VF can serve as a simple, non-invasive, and clinically applicable indicator for CVD risk stratification in this population. These findings suggest that monitoring cumulative metabolic burden in the early stages of CKM syndrome may help identify high-risk individuals and implement targeted interventions. Future studies are needed to validate these findings in diverse populations and further explore effective intervention strategies.

## Supplementary Information

Supplementary Material 1.

## Data Availability

The datasets supporting the conclusions of this article are publicly available in the CHARLS repository [http://charls.pku.edu.cn]. Access to the data requires registration, and data use is restricted to academic and research purposes only.

## Abbreviations

AHA: American heart association
ANOVA: Analysis of variance
BMI: Body mass index
BUN: Blood urea nitrogen
CHARLS: China health and retirement longitudinal study
CI: Confidence interval
CIs: Confidence intervals
CKD: Chronic kidney disease
CKD-EPI: Chronic kidney disease epidemiology collaboration
CKM: Cardiovascular–kidney–metabolic syndrome
cumMETS-VF: Cumulative metabolic score for visceral fat
CVD: Cardiovascular disease
DBP: Diastolic blood pressure
eGFR: Estimated Glomerular Filtration Rate
FBG: Fasting blood glucose
GLU: Glucose
HbA1c: Hemoglobin A1c
HDL-C: High-density lipoprotein cholesterol
HR: Hazard ratio
Hs-CRP: High-sensitivity C-reactive protein
IQR: Interquartile range
LDL-C: Low-density lipoprotein cholesterol
METS-IR: Metabolic score for insulin resistance
METS-VF: Metabolic score for visceral fat
Q: Quartile
RAAS: Renin-angiotensin-aldosterone system
RCS: Restricted cubic splines
SBP: Systolic blood pressure
Scr: Serum creatinine
SD: Standard deviation
TC: Total cholesterol
TG: Triglyceride
UA: Uric acid
WHtR: Waist-to-height ratio
