# Supplementary material for: Association between cumulative metabolic score for visceral fat and cardiovascular disease risk in cardiovascular-kidney-metabolic syndrome stage 0–3 patients: a prospective cohort analysis based on CHARLS database
Source: BMC Cardiovasc Disord. 2026 Mar 30;26:405. doi: 10.1186/s12872-026-05801-0 (PMC13154521; doi:10.1186/s12872-026-05801-0)
Supplement: Supplementary file 1 — Supplementary Material 1. [file 12872_2026_5801_MOESM1_ESM.docx]

**Supplemental materials**

**Table S1** Comparison of baseline characteristics between participants included in the analysis (n = 3,341) and those excluded primarily due to missing data on key variables (n = 11,821)

| **Characteristic** | **Enrollment participants N = 3,341** | **Excluded participants N = 11,821** | **p-value** |
| --- | --- | --- | --- |
| Male, n (%) | 1,566 (47) | 5730 (48) | 0.10 |
| Age(years) | 58 ± 9 | 58 ± 11 | <0.001 |
| Urban residence, n (%) | 448 (13) | 2858 (24) | <0.001 |
| BMI(kg/m²) | 23.4 | 24.2 | 0.13 |
| Dyslipidaemia, n (%) | 272 (8.1) | 1018(8.9) | 0.2 |
| Hypertension, n (%) | 714 (21) | 2855 (25) | <0.001 |
| Diabetes，n (%) | 186 (5.6) | 670(5.8) | 0.6 |
| Diabetes medication use, n (%) | 113 (3.4) | 459(4.0) | 0.12 |
| Diabetes insulin use, n (%) | 16 (0.5) | 94 (0.8) | 0.45 |
| Kidney disease, n (%) | 138 (4.1) | 635 (5.5) | 0.002 |
| Liver disease, n (%) | 91 (2.7) | 398 (3.4) | 0.051 |
| Lipid-lowering medication use, n (%) | 133 (4.0) | 503 (4.4) | 0.3 |
| Antihypertensive medication use, n (%) | 488 (15) | 2152 (19) | <0.001 |

**Note:** Excluded participants includes participants excluded from the analysis mainly due to missing data on biochemical measurements and other key variables required for exposure construction, covariate adjustment, or outcome assessment.

Table S2 Definitions of CKM.

| **CKM syndrome stages** | **Definition** |
| --- | --- |
| Stage 0: No CKM risk factors | Individuals with normal BMI and waist circumference, normoglycemia, normotension, a normal lipid profile, and no evidence of CKD or subclinical or clinical CVD. |
| Stage 1: Excess or dysfunctional adiposity | Individuals with overweight/obesity, abdominal obesity, or dysfunctional adipose tissue, without the presence of other metabolic risk factors or CKD. ①BMI ≥ 23 kg/m².  ②Waist circumference ≥ 80cm in women or ≥90cm in men. ③Fasting blood glucose ≥ 100–124 mg/dL, or HbA₁c between 5.7% and 6.4%*. |
| Stage 2: Metabolic risk factors and CKD | Individuals with metabolic risk factors (hypertriglyceridemia, hypertension, MetS# (see Table S2), diabetes), or moderate-to-high-risk CKD stage†.  ①Hypertriglyceridemia is defined by TG ≥ 135mg/dL.  ②Hypertension is defined by SBP ≥ 130 mmHg, DBP ≥ 80 mmHg, a medical diagnosis, or taking antihypertensive medication(1).  ③Diabetes is defined by FBG > 126 mg/dL, HbA₁c ≥ 6.5%, a medical diagnosis, or taking insulin or glucose-lowering medication(2).  ④Moderate-to-high-risk CKD: UACR ≥ 30 mg/g and eGFR ≥ 60 ml/min/1.73m2, UACR < 300 mg/g and eGFR ≤ 45-59 ml/min/1.73m2, or UACR < 30 mg/g and eGFR ≤ 30-44 ml/min/1.73m2. |
| Stage 3: Subclinical CVD‡ in CKM | Risk equivalents of subclinical CVD. In this study, among participants classified as CKM stage 3 (n = 349), 348 (99.7%) were categorized based on PREVENT-predicted 10-year CVD risk ≥20%, whereas only 1 (0.3%) met the KDIGO very high-risk CKD criteria. ①Very high-risk CKD in KDIGO classification: UACR ≥300 mg/g and eGFR ≤45–59 ml/min/1.73m2, UACR ≥30 mg/g and eGFR ≤30–44 ml/min/1.73m2, or eGFR ≤29 ml/min/1.73m2.  ②High predicted 10-year CVD risk: the predicted risk ≥20%, as calculated by the PREVENT equations§ (see Table S3)(3,4). |

**Note:**

The CKM staging framework was adapted from the AHA scientific statement on cardiovascular-kidney-metabolic health. Definitions of hypertension and diabetes were based on the 2017 ACC/AHA hypertension guideline and the American Diabetes Association Standards of Care, respectively. Predicted cardiovascular risk was estimated using the PREVENT equations.

* Gestational Diabetes: Individuals with gestational diabetes should receive intensified screening for impaired glucose tolerance after pregnancy.

# MetS: See Table S2 for detailed diagnostic criteria.

†The stage of CKD is determined by the KDIGO criteria, using eGFR and UACR(5). The eGFR was computed using the 2021 race and ethnicity-free CKD-EPI creatinine equation(6). Due to the absence of urine test data, CKD staging was based on eGFR alone.

‡Subclinical CVD was defined as the presence of risk equivalents indicating advanced cardiovascular damage.

§ PREVENT Equations: See Table S3 for detailed calculation method.

**Abbreviations:** BMI, body mass index; CKD, chronic kidney disease; CKD-EPI, Chronic Kidney Disease Epidemiology Collaboration; CKM, cardiovascular-kidney-metabolic; CVD, cardiovascular disease; DBP, diastolic blood pressure; eGFR, estimated glomerular filtration rate; FBG, fasting blood glucose; HbA₁c, hemoglobin A1c; HDL-C, high-density lipoprotein cholesterol; KDIGO, Kidney Disease: Improving Global Outcomes; MetS, metabolic syndrome; PREVENT, Predicting Risk of cardiovascular disease EVENTs; SBP, systolic blood pressure; TC, total cholesterol; TG, triglycerides; UACR, urine albumin-to-creatinine ratio.

Table S3 Metabolic Syndrome (MetS) Definition.

MetS is defined by the presence of 3 or more of the following criteria:

| **Component** | **Criteria** |
| --- | --- |
| (1) Waist Circumference* | • ≥ 88 cm for women or ≥ 102 cm for men (general population) • ≥ 80 cm for women or ≥ 90 cm for men (Asian ancestry) |
| (2) HDL Cholesterol | • < 40 mg/dL for men • < 50 mg/dL for women |
| (3) Triglycerides (TG) | ≥ 150 mg/dL |
| (4) Blood Pressure | SBP ≥ 130 mmHg or DBP ≥ 80 mmHg or use of antihypertensive medications |
| (5) Fasting Blood Glucose (FBG) | ≥ 100 mg/dL |

**Note:**

*For Asian populations in this study, the lower waist circumference cutoffs (≥80 cm for women and ≥90 cm for men) were applied.

Table S4 The PREVENT 10-year Risk Estimation Model Equations.

| **Sex** | **Calculation** |
| --- | --- |
| Men | log-Odds = -3.031168 + 0.7688528 × (age - 55) /10 + 0.0736174 × ((TC - HDL-C) × 0.02586 - 3.5) - 0.0954431 × (HDL-C × 0.02586 - 1.3) /0.3 - 0.4347345 × (min(SBP, 110) - 110) /20 + 0.3362658 × (max(SBP, 110) - 130) /20 + 0.7692857 × (if diabetes) + 0.4386871 × (if current smoker) + 0.5378979 × (min(eGFR, 60) - 60) / -15 + 0.0164827 × (max(eGFR, 60) - 90) / -15 + 0.288879 × (if using anti-hypertensive medication) - 0.1337349 × (if using statin) - 0.0475924 × (if using anti-hypertensive medication) × (max(SBP, 110) - 130) /20 + 0.150273 × (if using statin) × ((TC - HDL-C) × 0.02586 - 3.5) - 0.0517874 × (age - 55) /10 × ((TC - HDL-C) × 0.02586 - 3.5) + 0.0191169 × (age - 55) /10 × (HDL-C × 0.02586 - 1.3) /0.3 - 0.1049477 × (age - 55) /10 × (max(SBP, 110) - 130) /20 - 0.2251948 × (age - 55) /10 × (if diabetes) - 0.0895067 × (age - 55) /10 × (if current smoker) - 0.1543702 × (age - 55) /10 × (min(eGFR, 60) - 60) / -15 |
|  | Risk = 1 / (1 + exp(-log-Odds)) |
| Women | log-Odds = -3.307728 + 0.7939329 × (age - 55) /10 + 0.0305239 × ((TC - HDL-C) × 0.02586 - 3.5) - 0.1606857 × (HDL-C × 0.02586 - 1.3) /0.3 - 0.2394003 × (min(SBP, 110) - 110) /20 + 0.360078 × (max(SBP, 110) - 130) /20 + 0.8667604 × (if diabetes) + 0.5360739 × (if current smoker) + 0.6045917 × (min(eGFR, 60) - 60) / -15 + 0.0433769 × (max(eGFR, 60) - 90) / -15 + 0.3151672 × (if using anti-hypertensive medication) - 0.1477655 × (if using statin) - 0.0663612 × (if using anti-hypertensive medication) × (max(SBP, 110) - 130) /20 + 0.1197879 × (if using statin) × ((TC - HDL-C) × 0.02586 - 3.5) - 0.0819715 × (age - 55) /10 × ((TC - HDL-C) × 0.02586 - 3.5) + 0.0306769 × (age - 55) /10 × (HDL-C × 0.02586 - 1.3) /0.3 - 0.0946348 × (age - 55) /10 × (max(SBP, 110) - 130) /20 - 0.27057 × (age - 55) /10 × (if diabetes) - 0.078715 × (age - 55) /10 × (if current smoker) - 0.1637806 × (age - 55) /10 × (min(eGFR, 60) - 60) / -15 |
|  | Risk = 1 / (1 + exp(-log-Odds)) |

**References**

1. Whelton PK, Carey RM. The 2017 American college of cardiology/american heart association clinical practice guideline for high blood pressure in adults. JAMA Cardiol. 2018 Apr 1;3(4):352-3.

2. American Diabetes Association Professional Practice Committee. 2. Classification and diagnosis of diabetes: Standards of medical care in diabetes-2022. Diabetes Care. 2022 Jan 1;45(Suppl 1):S17-38.

3. Khan SS, Coresh J, Pencina MJ, Ndumele CE, Rangaswami J, Chow SL, et al. Novel prediction equations for absolute risk assessment of total cardiovascular disease incorporating cardiovascular-kidney-metabolic health: A scientific statement from the American heart association. Circulation. 2023 Dec 12;148(24):1982-2004.

4. Khan SS, Matsushita K, Sang Y, Ballew SH, Grams ME, Surapaneni A, et al. Development and validation of the American heart association’s PREVENT equations. Circulation. 2024 Feb 6;149(6):430-49.

5. Kidney Disease: Improving Global Outcomes (KDIGO) CKD Work Group. KDIGO 2024 clinical practice guideline for the evaluation and management of chronic kidney disease. Kidney Int. 2024 Apr;105(4S):S117-314.

6. Inker LA, Eneanya ND, Coresh J, Tighiouart H, Wang D, Sang Y, et al. New creatinine- and cystatin C-based equations to estimate GFR without race. N Engl J Med. 2021 Nov 4;385(19):1737-49.

**Table S5** Schoenfeld residual test for proportional hazards assumption.

| Variable | Per SD model (p) | Quartile model (p) | Trend model (p) |
| --- | --- | --- | --- |
| cumMETSVF | 0.1586 | 0.7512 | 0.4870 |
| Systolic BP | 0.0071 | 0.0069 | 0.0070 |
| Diastolic BP | 0.0230 | 0.0222 | 0.0220 |
| Fasting glucose | 0.0469 | 0.0446 | 0.0450 |
| GLOBAL | 0.0912 | 0.1723 | 0.1200 |

**Notes:**

P values were obtained from Schoenfeld residual tests. A p-value <0.05 indicates potential violation of the proportional hazards assumption. The global tests were not significant, suggesting no overall violation.

**Table S6** Collinearity Statistics.

| Variable | GVIF | DF | GVIF^(1/(2*Df)) |
| --- | --- | --- | --- |
| cumMETSVF | 1.0547 | 1 | 1.0270 |
| Age | 1.2942 | 1 | 1.1376 |
| Gender | 2.4507 | 1 | 1.5655 |
| Marital status | 1.0678 | 1 | 1.0333 |
| Educational level | 2.5489 | 2 | 1.5965 |
| Residence | 1.1587 | 1 | 1.0764 |
| Smoking status | 1.9568 | 2 | 1.3988 |
| Drinking status | 1.4687 | 2 | 1.2119 |
| Hypertension | 1.9831 | 1 | 1.4082 |
| Dyslipidemia | 1.7146 | 1 | 1.3094 |
| Diabetes | 2.8248 | 1 | 1.6807 |
| Kidney disease | 1.0188 | 1 | 1.0094 |
| Liver disease | 1.0233 | 1 | 1.0116 |
| Antihypertensive medication use | 2.0351 | 1 | 1.4266 |
| Lipid-lowering medication use | 1.6955 | 1 | 1.3021 |
| Diabetes medication use | 1.3439 | 1 | 1.1593 |
| Diabetes insulin use | 1.2594 | 1 | 1.1222 |
| Hs-CRP | 1.0129 | 1 | 1.0064 |
| LDL-C | 1.0418 | 1 | 1.0207 |

**Notes:**

generalized variance inflation factor; Df: degrees of freedom. GVIF^(1/(2×Df)) was used to assess multicollinearity. For variables with multiple degrees of freedom (e.g., educational level, smoking status, and drinking status), the largest GVIF and corresponding GVIF^(1/(2×Df)) were reported.

**Figure S1.** Kaplan-Meier Curves for CVD-Free Survival by CumMETS-VF Quartiles (Cancer Cases Excluded)
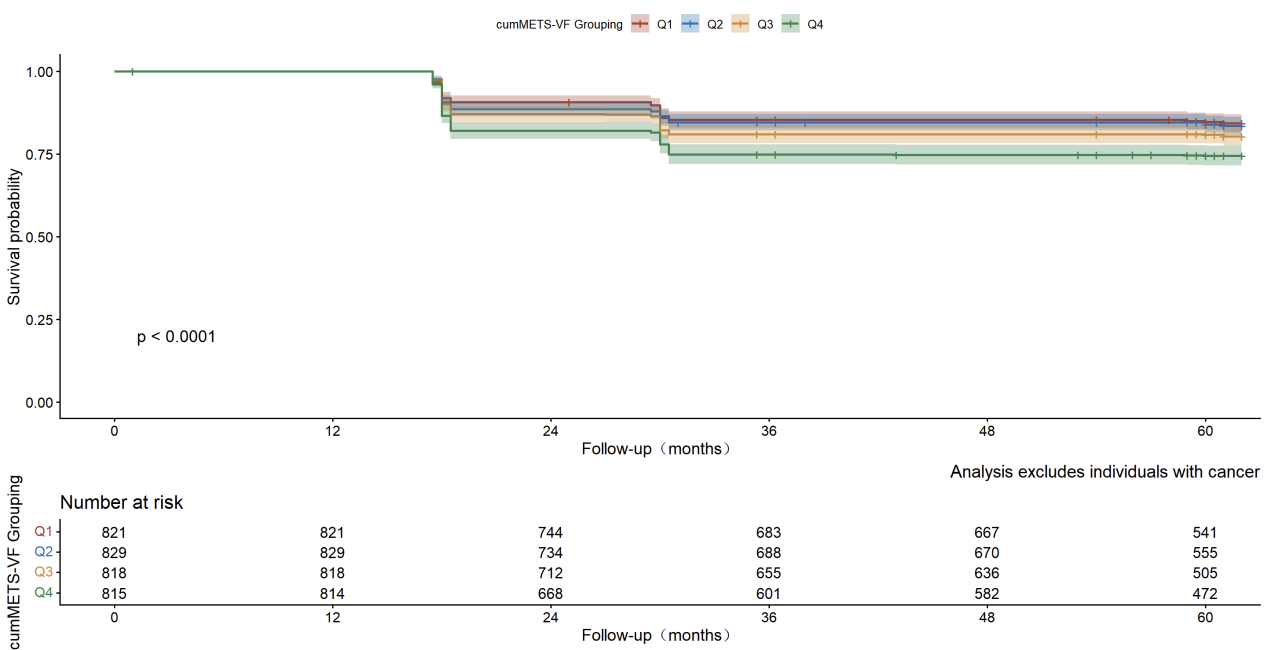


Kaplan-Meier survival curves showing cumulative CVD-free survival rates stratified by cumMETS-VF exposure quartiles (Q1-Q4) in individuals with CKM syndrome stages 0-3, with cancer cases excluded from the analysis. The log-rank test indicated significant differences between groups (P <0.0001). This sensitivity analysis demonstrates the robustness of the main findings when cancer cases are removed, confirming that the association between cumMETS-VF and CVD risk remains statistically significant independent of cancer history.

**Figure S2.** Dose-Response Relationship Between CumMETS-VF Exposure and CVD Risk (Cancer Cases Excluded).


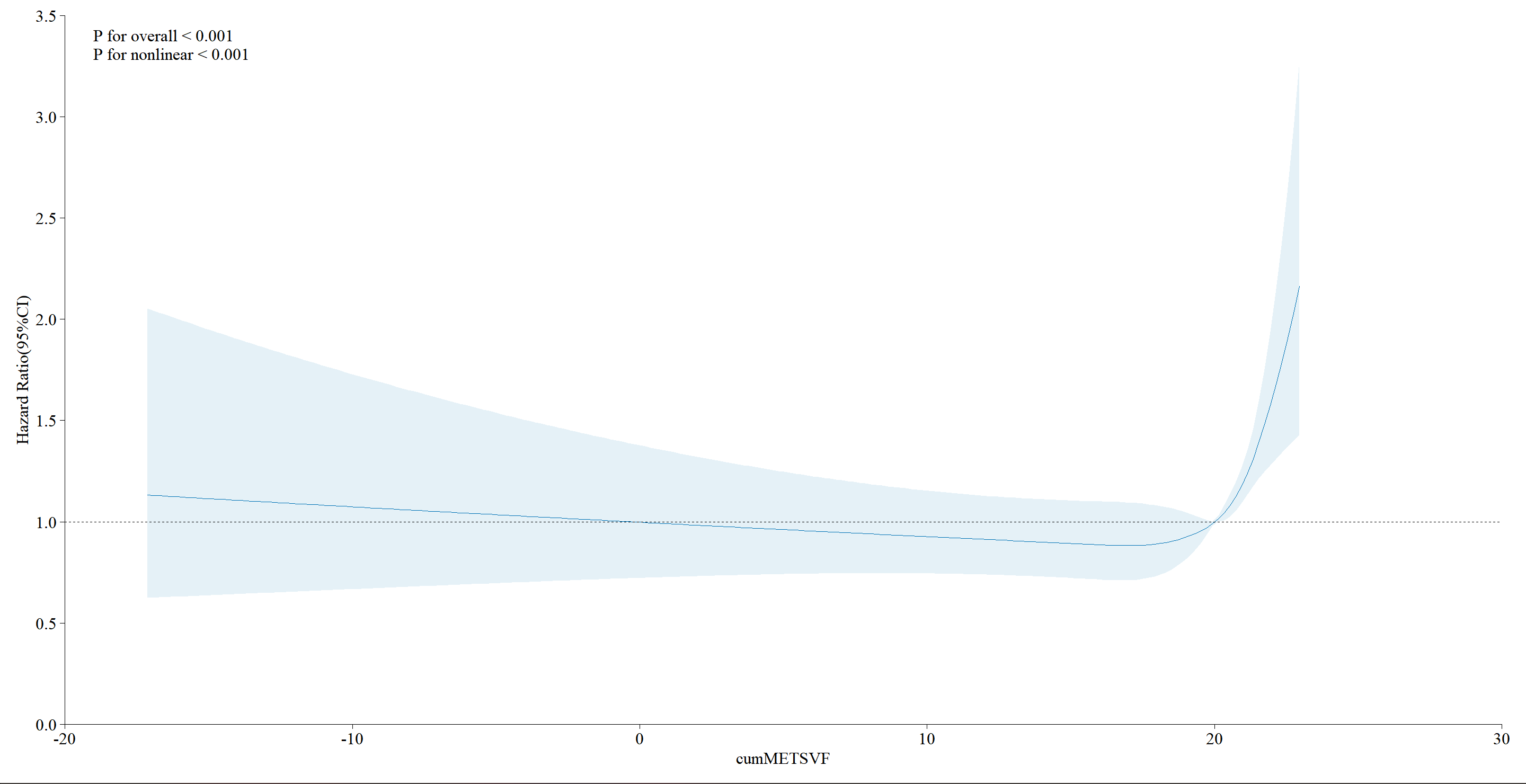


Restricted cubic spline curves illustrating the dose-response relationship between cumMETS-VF exposure and CVD risk with cancer cases excluded from the analysis. Overall analysis used 4 knots at the 5th, 35th, 65th, and 95th percentiles. The reference point (HR =1.0) is set at the median cumMETS-VF. Solid lines represent hazard ratios (HRs); shaded areas represent 95% confidence intervals (CIs). P values indicate overall association (P for overall) and non-linearity (P for nonlinearity). This sensitivity analysis confirms the robustness of the dose-response relationship when cancer cases are excluded from the analysis.

Table S7 Association of Cumulative Exposure to METS-VF with the Risk of CVD in a CKM Syndrome Stage 0-3 Population After Excluding Individuals With Any Missing Data or Cancer.

|  |  | HR (95% CI) | | |
| --- | --- | --- | --- | --- |
| Quartile | Cases/Total | Model 1 | Model 2 | Model 3 |
| Q1 (Ref) | 126 / 821 | Reference | Reference | Reference |
| Q2 | 133 / 829 | 1.03 (0.81, 1.31) | 1.01 (0.79, 1.29) | 1.00 (0.78, 1.28) |
| Q3 | 158 / 818 | 1.23 (0.97, 1.55) | 1.16 (0.92, 1.48) | 1.09 (0.86, 1.40) |
| Q4 | 208 / 815 | 1.70 (1.36, 2.12) | 1.52 (1.20, 1.92) | 1.31 (1.02, 1.69) |
| P for trend |  | <0.001 | <0.001 | <0.001 |
| Events/N | 625/ 3283 |  |  |  |

**Notes:**

Model 1: Adjusted for age and sex.

Model 2: Model 1 + marital status, education, residential area, smoking, drinking, systolic blood pressure, diastolic blood pressure, fasting blood glucose, and HbA1c.

Model 3: Model 2 + total cholesterol, HDL, eGFR, history of dyslipidemia, hypertension, diabetes, and medications for dyslipidemia, hypertension, and diabetes.

Events/N: Number of CVD events / Total number of participants.

Table S8 Subgroup Analyses of the Association Between Cumulative Exposure to METS-VF and the Risk of CVD in a CKM Syndrome Stage 0-3 Population After Excluding Individuals With Any Missing Data or Cancer.

| **Variable** | | **Count** | **Percent（%）** | **HR (95% CI)** | **P value** | **P for interaction** |
| --- | --- | --- | --- | --- | --- | --- |
| Overall |  | 3283 | 100 | 1.13 (1.05–1.22) | 0.001 | - |
| Age | <60 | 1931 | 58 | 1.13 (1.02–1.26) | 0.019 | 0.603 |
|  | ≥60 | 1352 | 41 | 1.12 (1.01–1.25) | 0.036 | - |
| Gender | Females | 1739 | 53 | 1.11 (1.01–1.23) | 0.039 | 0.644 |
|  | Males | 1544 | 47 | 1.15 (1.03–1.28) | 0.016 | - |
| Smoking | Current Smoker | 1024 | 31 | 1.10 (0.96–1.27) | 0.155 | 0.944 |
|  | Former Smoker | 238 | 7 | 1.20 (0.91–1.58) | 0.201 | - |
|  | Never Smoked | 2021 | 61 | 1.13 (1.03–1.25) | 0.011 | - |
| Drinking | Current Drinker | 1074 | 32 | 1.17 (1.02–1.34) | 0.026 | 0.726 |
|  | Former Drinker | 243 | 7 | 1.05 (0.83–1.34) | 0.671 | - |
|  | Never Drinker | 1966 | 59 | 1.13 (1.02–1.24) | 0.014 | - |
| Dyslipidemia | No | 3036 | 92 | 1.14 (1.05–1.23) | 0.002 | 0.714 |
|  | Yes | 247 | 7 | 1.08 (0.87–1.34) | 0.467 | - |
| Hypertension | No | 2580 | 78 | 1.11 (1.01–1.21) | 0.023 | 0.532 |
|  | Yes | 703 | 21 | 1.20 (1.03–1.40) | 0.019 | - |
| Diabetes | No | 3102 | 94 | 1.14 (1.06–1.24) | 0.001 | 0.208 |
|  | Yes | 181 | 5 | 1.01 (0.77–1.33) | 0.943 | - |
| CKM | 0 | 356 | 10 | 1.07 (0.60–1.93) | 0.811 | 0.026 |
|  | 1 | 824 | 25 | 1.11 (0.94–1.30) | 0.215 | - |
|  | 2 | 1758 | 53 | 1.07 (0.97–1.18) | 0.184 | - |
|  | 3 | 345 | 10 | 1.61 (1.18–2.20) | 0.003 | - |

**Figure S3.** Kaplan-Meier Curves for CVD-Free Survival by CumMETS-VF Quartiles (Diabetes Medications Excluded)

**
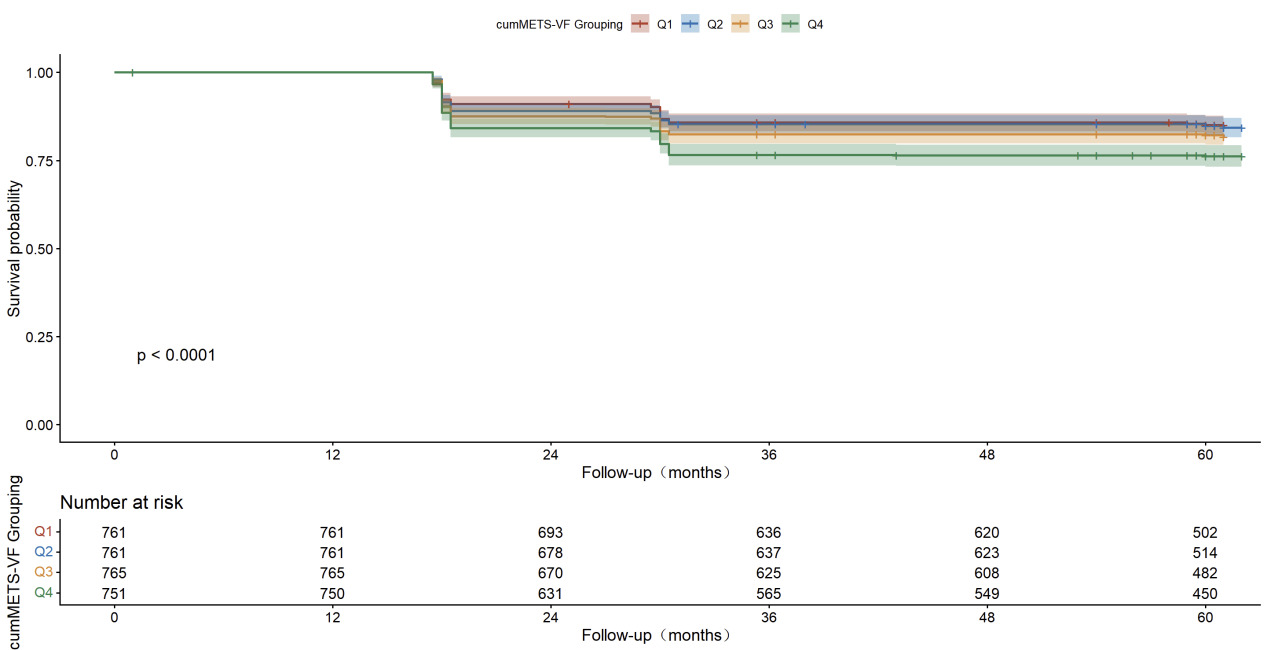
**

Kaplan-Meier survival curves showing cumulative CVD-free survival rates stratified by cumMETS-VF exposure quartiles (Q1-Q4) in participants not taking diabetes medications. The log-rank test indicated significant differences between groups (P <0.0001). This sensitivity analysis excludes participants taking diabetes medications to evaluate the association between cumMETS-VF and CVD risk independent of diabetes medication use, demonstrating that the association remains significant even when medication use is controlled for.

**Figure S4.** Dose-Response Relationship Between CumMETS-VF Exposure and CVD Risk (Diabetes Medications Excluded
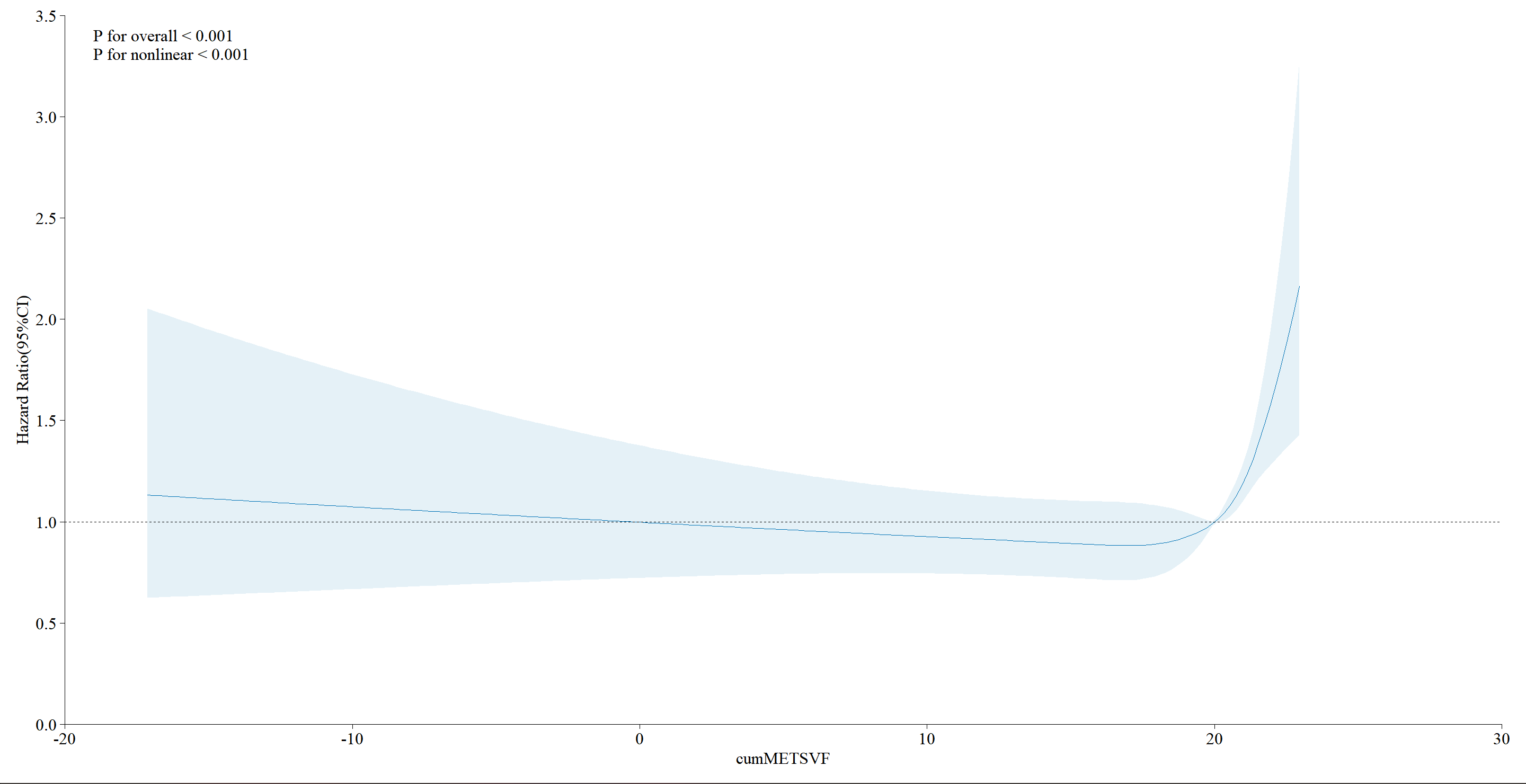


**Note:**Restricted cubic spline curves showing the dose-response relationship between cumMETS-VF exposure and CVD risk with participants taking diabetes medications excluded from the analysis. Overall analysis used 4 knots at the 5th, 35th, 65th, and 95th percentiles. The reference point (HR =1.0) is set at the median cumMETS-VF. Solid lines represent hazard ratios (HRs); shaded areas represent 95% confidence intervals (CIs). P values indicate overall association and non-linearity. This sensitivity analysis confirms that the observed dose-response relationship between cumMETS-VF and CVD risk is independent of diabetes medication use.

Table S9 Association of Cumulative Exposure to METS-VF with the Risk of CVD in a CKM Syndrome Stage 0-3 Population After Excluding Individuals With Any Missing Data and Diabetes Medication Use.

|  |  | HR (95% CI) | | |
| --- | --- | --- | --- | --- |
| Quartile | Cases/Total | Model 1 | Model 2 | Model 3 |
| Q1 (Ref) | 113 / 761 | Reference | Reference | Reference |
| Q2 | 116 / 761 | 1.013 (0.781–1.314) | 1.000 (0.770–1.298) | 0.998 (0.768–1.298) |
| Q3 | 137 / 765 | 1.188 (0.925–1.525) | 1.147 (0.891–1.478) | 1.093 (0.844–1.416) |
| Q4 | 178 / 751 | 1.596 (1.258–2.025) | 1.474 (1.148–1.893) | 1.325 (1.014–1.730) |
| P for trend |  | <0.001 | <0.001 | <0.001 |
| Events/N | 544/3038 |  |  |  |

**Notes:**

Model 1: Adjusted for age and sex.

Model 2: Model 1 + marital status, education, residential area, smoking, drinking, systolic blood pressure, diastolic blood pressure, fasting blood glucose, and HbA1c.

Model 3: Model 2 + total cholesterol, HDL, eGFR, history of dyslipidemia, hypertension, diabetes, and medications for dyslipidemia, hypertension, and diabetes.

Events/N: Number of CVD events / Total number of participants.

Table S10 Subgroup Analyses of the Association Between Cumulative Exposure to METS-VF and the Risk of CVD in a CKM Syndrome Stage 0-3 Population After Excluding Individuals With Any Missing Data and Diabetes Medication Use.

| **Variable** | | **Count** | **Percent（%）** | **HR (95% CI)** | **P value** | **P for interaction** |
| --- | --- | --- | --- | --- | --- | --- |
| Overall |  | 3038 | 100 | 1.13 (1.04–1.22) | 0.003 | - |
| Age | <60 | 1805 | 59.4 | 1.12 (1.00–1.26) | 0.042 | 0.91 |
|  | ≥60 | 1233 | 40.6 | 1.13 (1.01–1.26) | 0.04 | - |
| Gender | Females | 1587 | 52.2 | 1.10 (0.98–1.22) | 0.104 | 0.445 |
|  | Males | 1451 | 47.8 | 1.15 (1.03–1.29) | 0.016 | - |
| Smoking | Current Smoker | 965 | 31.8 | 1.08 (0.94–1.25) | 0.272 | 0.683 |
|  | Former Smoker | 219 | 7.2 | 1.29 (0.95–1.73) | 0.098 | - |
|  | Never Smoked | 1854 | 61.0 | 1.13 (1.02–1.25) | 0.021 | - |
| Drinking | Current Drinker | 1013 | 33.3 | 1.10 (0.95–1.27) | 0.191 | 0.945 |
|  | Former Drinker | 215 | 7.1 | 1.16 (0.90–1.51) | 0.257 | - |
|  | Never Drinker | 1810 | 59.6 | 1.14 (1.03–1.26) | 0.014 | - |
| Dyslipidemia | No | 2838 | 93.4 | 1.13 (1.04–1.23) | 0.005 | 0.876 |
|  | Yes | 200 | 6.6 | 1.10 (0.85–1.41) | 0.477 | - |
| Hypertension | No | 2440 | 80.3 | 1.10 (1.01–1.21) | 0.038 | 0.43 |
|  | Yes | 598 | 19.7 | 1.22 (1.03–1.44) | 0.02 | - |
| Diabetes | No | 3019 | 99.4 | 1.13 (1.04–1.22) | 0.003 | 0.874 |
|  | Yes | 19 | 0.6 | 0.00 (0.00–0.00) | <0.001 | - |
| CKM | 0 | 354 | 11.7 | 1.04 (0.59–1.83) | 0.893 | 0.14 |
|  | 1 | 806 | 26.5 | 1.09 (0.92–1.29) | 0.316 | - |
|  | 2 | 1607 | 52.9 | 1.09 (0.98–1.21) | 0.117 | - |
|  | 3 | 271 | 8.9 | 1.47 (1.05–2.04) | 0.023 | - |
